# Platelet-rich plasma for the treatment of chronic rectal ulcer: A case report

**DOI:** 10.1097/MD.0000000000030016

**Published:** 2022-10-14

**Authors:** Gengjun Liu, Ying Li, Yaxin Li, Licun Wang, Ping Li, Zheng Liu, Jiao Liu, Dongmei Ge, Gang Zhao, Haiyan Wang

**Affiliations:** a Department of Blood Transfusion, the Affiliated Hospital of Qingdao University, Qingdao 266000, Shandong, China; b Department of Anorectal Surgery, the Affiliated Hospital of Qingdao University, Qingdao 266000, Shandong, China.

**Keywords:** case report, platelet-rich plasma (PRP), rectal ulcer, wound healing

## Abstract

Rectum ulcer is a disease of the digestive system, the main symptoms of which includes bloody diarrhea, abdominal pain, hematochezia, etc. At present, drug therapy and surgery are the most common treatments. Platelet-rich plasma (PRP) contains high concentrations of platelets and has been used to promote wound healing. However, the utilization of PRP in rectal ulcers has rarely been reported.

**Patient concerns:**

The patient had reported a complaint of blood dripping from the stool for more than 4 months. She had a history of surgery for rectal cancer with postoperative chemotherapy and radiotherapy 19 years prior. Mesalazine suppository was given to her for about 4 months, and glutamine capsules for 2 months, but the rectal ulcer remained unhealed.

**Diagnosis:**

A rectal ulcer was observed on colonoscopy, and the biopsy result was tubular adenoma.

**Interventions:**

Autologous PRP treatment was performed for the patient under an anorectal scope together with basic supportive care.

**Outcomes:**

The ulcer nearly healed within 9 days after twice PRP treatments.

**Lessons:**

PRP treatment may bring about novel treatment options for rectal ulcers.

## 1. Introduction

Rectal ulcer is a disease with the main symptoms of bloody diarrhea, abdominal pain, hematochezia, and weight loss. In addition to solitary rectal ulcers, rectal ulcer can be observed as a symptom in ulcerative colitis, rectal tumors, and ischemic bowel disease, while acute hemorrhagic rectal ulcers, radiation proctitis as well as infectious and drug-induced ones have also been reported.^[1–5]^ At present, topical therapy is the first choice for most patients. The effectiveness of medications, including sulfasalazine, mesalazine, corticosteroids, and sucralfate, has been proven.^[6,7]^ In addition, behavioral therapy such as biofeedback treatment appears to be promising for solitary rectal ulcers.^[7]^ Nevertheless, in some cases, conservative treatment is inefficacious. When deterioration or operative indications occur, surgery may be needed. If the ulcer becomes chronic, patients will end up with worse quality of life, longer hospital stays, and a heavier financial burden, and may eventually experience more physical and mental pain. As a result, more exploration and research should be carried out for the optimal treatment of chronic rectal ulcers.

The process of wound healing in skin and mucous membranes is complicated, and the crucial role that platelets play cannot be denied.^[8]^ There are a large number of alpha-granules in platelets, which contain growth factors, cytokines, and extracellular matrix regulators that play essential roles in hemostasis and tissue regeneration. After platelets are activated, the abovementioned bioactive proteins can be released, which promotes wound repair.^[9]^ Platelet-rich plasma (PRP) is a biological product that is defined as a portion of the plasma from autologous blood with a platelet concentration above baseline.^[10]^ Due to the extraction and concentration process, the platelet concentration of PRP is usually 5 times higher than the basal level.^[11]^ Before administration, activators such as calcium or thrombin are added to create activated autologous PRP,^[12]^ which leads to platelet aggregation and the release of alpha-granules, and PRP can be turned into platelet gel (PG). As an important source of growth factors for many treatments, PRP has been utilized widely in clinical therapy, including oral medicine and maxillofacial surgery, ophthalmology, orthopedics and dermatology, and more reports on wound healing have been noticed recently.^[12,13]^ However, the utilization of PRP in rectal ulcers has rarely been reported. In this case, PRP was utilized in the treatment of rectal ulcer and excellent effects were observed.

## 2. Case presentation

### 2.1. Patient

**Figure 1.**
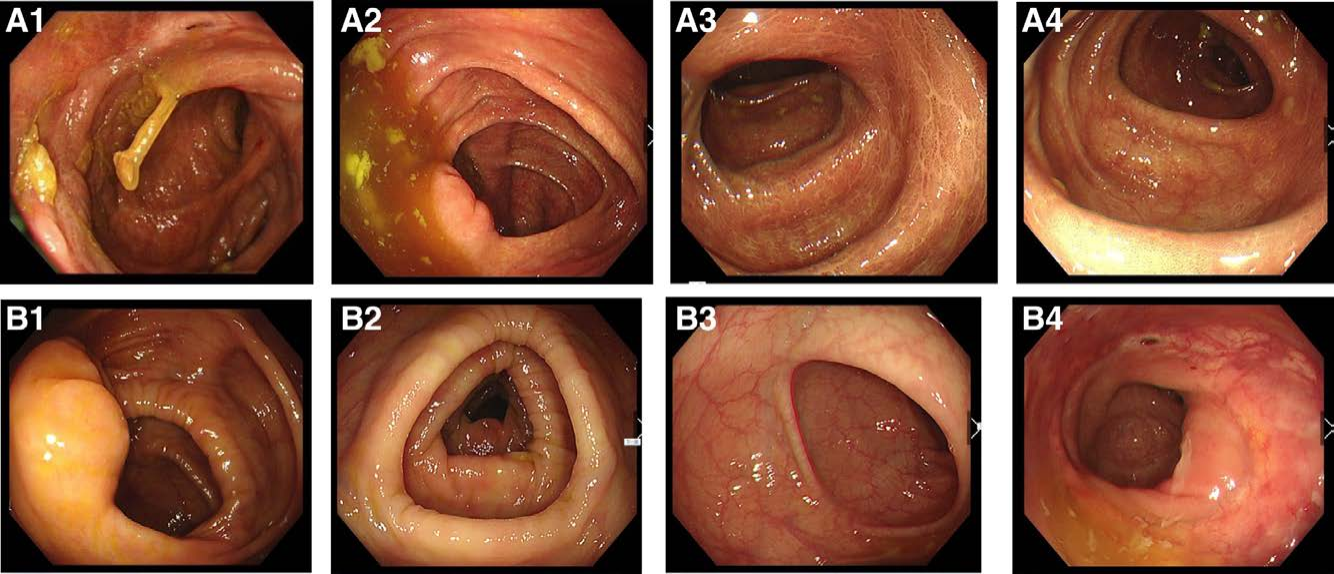
Pictures under colonoscopy. No sign of relapse or local ulcer on the surgical anastomosis site was found. (A) Date: March 24, 2018. (B) Date: February 28, 2019.

**Figure 2.**
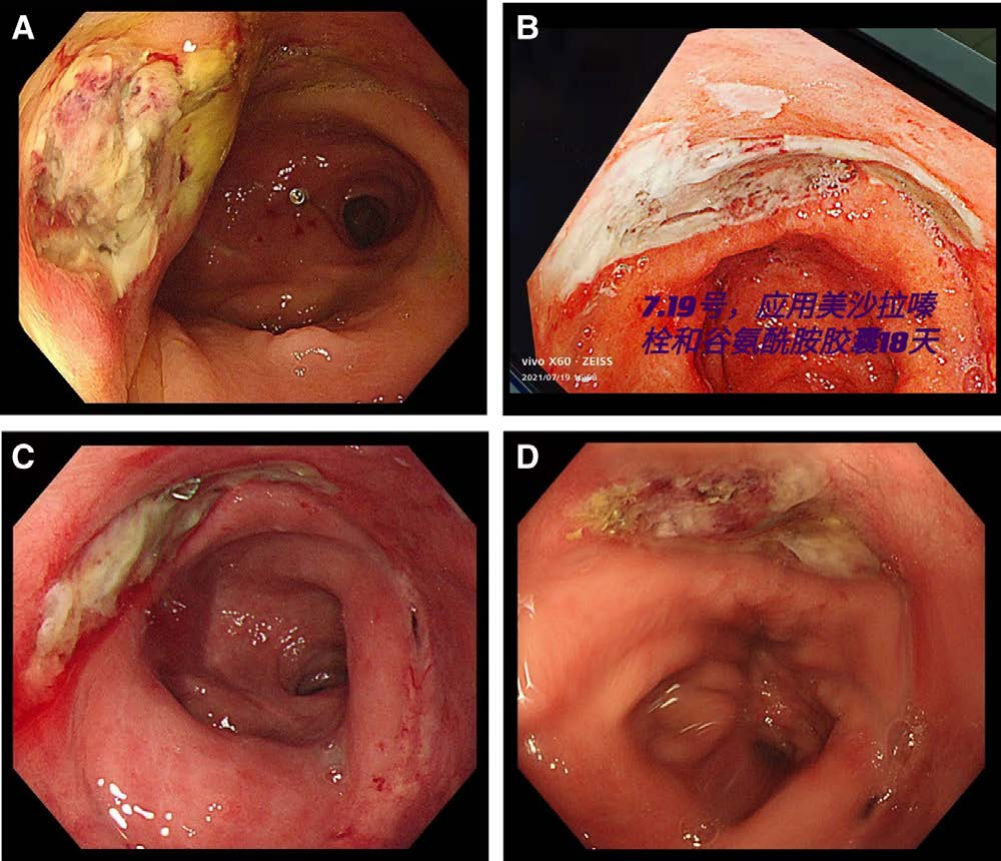
Pictures under colonoscopy. (A) Before treatment, June 22, 2021. (B) Mesalazine suppository treatment for 18 days, July 19, 2021. (C) Mesalazine suppository treatment for 28 days, August 12, 2021. (D) Mesalazine suppository treatment for approximately 3 and a half months, October 13, 2021.

A 70-year-old female patient presented for treatment with a chief complaint of blood dripping from stool on November 11, 2021. The medical history of this patient was complicated. Nineteen years prior, she underwent surgery for rectal cancer with postoperative chemotherapy and radiotherapy for 6 times and 25 times, respectively. Because of the time passed by, the discharge notes and details of that treatment could not be fully recalled. A colonoscopy examination had been performed twice respectively, on March 24, 2018 and February 28, 2019, and no sign of relapse or local ulcer on the surgical anastomosis site was found (Fig. 1A, B). Meanwhile, she had a history of hypertension, diabetes, coronary heart disease and severe osteoporosis for more than ten years, and medicine had been taken regularly. A history inquiry revealed that more than 4 months ago, the patient was hospitalized for the same reason. Colonoscopy on June 22, 2021 revealed a large ulcer with a diameter of approximately 2 × 3 cm at the anastomosis 3 cm from the anus (Fig. 2A). Biopsy was taken under colonoscopy, and the result was tubular adenoma. Due to inadequate surgical indications and a lack of intention for surgery, conservative medication was chosen. Mesalazine suppository was given to her for about 4 months, and glutamine capsules for 2 months. Since the treatment had been administered continuously for 18 days, 42 days, and 3 and a half months, the patient underwent colonoscopy respectively. After 18 days of treatment, the diameter of the ulcer was as large as the previous time (Fig. 2B). After 42 days of treatment, the diameter of the ulcer was 2 × 3.5 cm (Fig. 2C). At approximately 3 and a half months later, the diameter of the ulcer was 1 × 2 cm, which showed a modest decrease, but the ulcer remained unhealed (Fig. 2D). No lumps were touched in anal finger examination. Blood examination showed that coagulation indicators remained in the normal range, and a slight rise in her hepatic parameters (glutamic oxaloacetic transaminase or GOT, glutamic pyruvic transaminase or GPT, and bilirubin). No abnormality was found in routine stool examination.

### 2.2. PRP treatment

**Figure 3.**
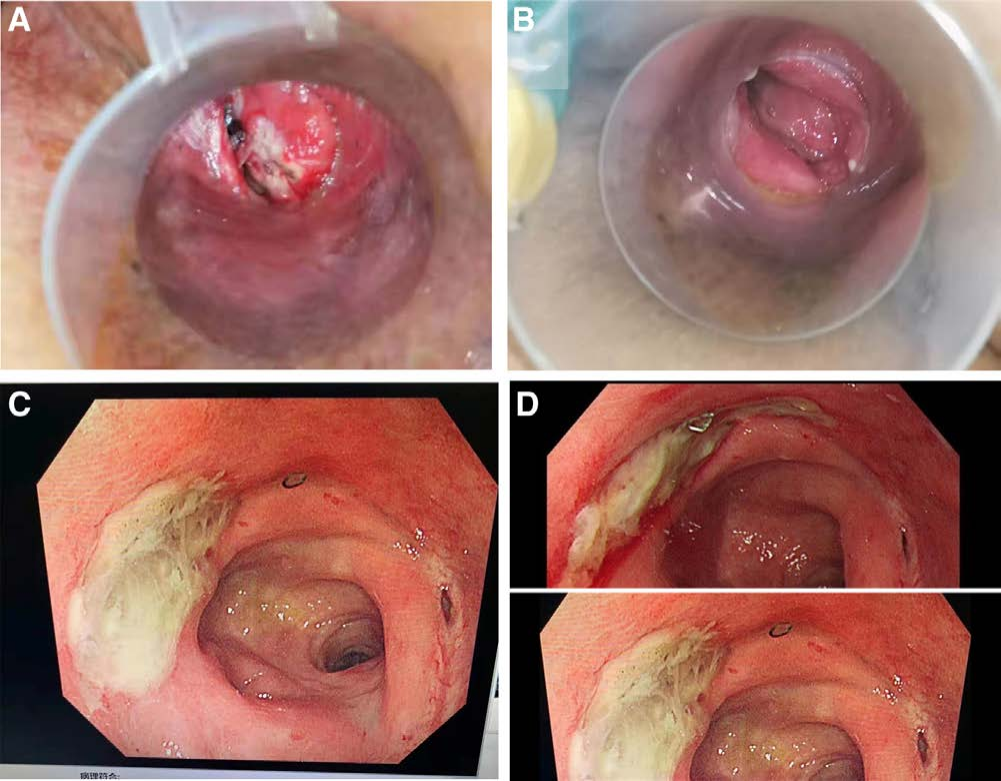
(A) A picture under anorectal scope. After the first PRP treatment, November 5, 2021. (B) A picture under anorectal scope. After the second PRP treatment, November 8, 2021. (C) Pictures under colonoscopy. After the second PRP treatment, November 24, 2021. Pus mosses can be seen near the anastomosis. (D) Comparison before and after PRP treatment.

Considering that the ulcer remained unhealed for more than 4 months, PRP treatment was suggested. After an all-sided consultation of the pros and cons, the patient agreed to receive the PRP treatment and informed consent was signed. Whole blood (150 mL) was drawn by venipuncture from the antecubital vein of the patient’s right arm, and was collected in a sterile triple blood bag that precontained 3.8% sodium citrate. Whole blood was initially centrifuged (1300 rpm for 10 minutes) to separate plasma from the red blood cell fraction (Hitachi CR7; Japan). The remaining PRP and PPP (platelet-poor plasma) portions were again centrifuged (3200 rpm for 15 minutes) to separate the PRP from the PPP. The obtained PRP was left at room temperature for 1–2 hours before treatment to get the platelets back in suspension. The operation process was performed in the clean bench, and the blood was always kept in a sterile sealed blood storage bag. The concentration of the prepared PRP was counted to be 723 × 10^11^/L (Mindray BC-2600; China). The whole administration process was carried out with anorectal scope. After debridement, PRP and activator (thrombin and calcium chloride) were co-sprayed onto the ulcer surface. When the PRP turned into PG, covering layer 1 was successfully created. Then the PRP and abovementioned activators were co-sprayed again onto the surgical gauze, which created PG gauze. It was applied to cover the ulcer surface and superimposed on the PG layer, giving rise to the covering layer 2. Finally, oil gauze was utilized for stuffing and fixation. The activator we used was 100 units of thrombin calcium chloride, and the injection was performed with an activator to PRP volume ratio of 1:10. In addition to local treatment, improved circulation and prophylactic antibiotic therapy were also given to the patient, and erythrocytes and PPP were transfused back to her. The patient did not complain about any discomfort during PRP treatment. Dressing change occurred 4 days later (November 5, 2021). Under the anorectal scope, we observed that the diameter of the ulcer diminished notably (Fig. 3A). The second PRP treatment was given on that day in the same way. Three days later (November 8, 2021), anorectal scope was performed again and the ulcer almost disappeared (Fig. 3B). Colonoscopy was also performed on November 24, 2021, healing of the ulcer on the anastomosis was distinctly observed (Fig. 3C, there were pus mosses near the anastomosis), especially when compared with the pictures taken under colonoscopy before PRP treatment (Fig. 3D). The patient showed satisfaction about the result.

## 3. Discussion and Conclusions

As mentioned in the introduction, PRP has been widely used in clinical practice and has been proven to be remarkably effective. The predominant regeneration effect makes it a promising candidate for chronic wound healing. Chronic unhealing wounds are more likely to occur in patients with diabetes or immunological defects such as acquired immunodeficiency syndrome, or in patients with extensive traumatic lesions and burns.^[14]^ There have been quite a few published clinical and experimental reports that PRP can be applied in the treatment for patients with chronic cutaneous ulcers, and good results have been achieved.^[12,15]^ Additionally, due to the process of separation from autologous blood, PRP poses a low risk of adverse reactions such as immune rejection and allergies. Although the safety and efficacy of PRP have not been fully confirmed by large-scale clinical trials so far, and reports of adverse reactions can be seen occasionally,^[16]^ its enormous potential in regenerative medicine cannot be denied. The above are the reasons why PRP treatment was chosen in this case.

In the treatment of rectal ulcers, conservative medication remains the choice for most patients. Although sulfasalazine, mesalazine, and corticosteroids are the most common medicines for topical therapy, and their therapeutic effect has been reported in some cases, further evaluation is needed to determine the response rate and long-term effectiveness.^[7]^ And surgery is a necessary choice for patients with surgical indications. In this case, the patient had been suffering from chronic rectal ulcer for more than 4 months. Traditional topical therapy had some effect on her, but was not strong enough to cure the ulcer. In contrast, the ulcer almost healed within 9 days after 2 PRP treatments. From this observation, we maintain that PRP treatment may be the better option for patients with chronic unhealing rectal ulcers who are not sensitive to conservative medication and at the same time unwilling to receive surgery.

Meanwhile, there are still some issues in this case that we need to think over. Firstly, because of the limited number of patients, limitations cannot be avoided in this study. Furthermore, when we make good use of the excellent regeneration effect of PRP, the adverse effect it may bring about should also be considered. The local biochemical events that take place in healing of chronic rectal ulcer have not been well defined. When high concentration of platelet product is locally utilized, platelet-derived cytokines and growth factors would be released, which not only play a significant role in wound healing but also contribute to the stimulation of proliferation. What we need to think about is that, when the patient has the precancerous lesions or the medical history of tumor, whether the local application of PRP would promote tumorigenesis. It has been reported that the crosstalk between platelet and tumor is complicated. Platelets can activate tumor invasion and support metastasis, as well as promote tumor evasion of immune destruction.^[17]^ But there is still no strong evidence to prove that PRP can promote tumor at present. On the contrary, PRP may be a good helper in treatment for patient with tumor. Dias LP et al proposed that PRP in combination with Bacillus Calmette-Guerin immunotherapy can be used for the treatment of bladder cancer.^[18]^ With the combination of laser phototherapy, PRP was also proved to improve healing of osteonecrosis of the jaws in cancer patients.^[19]^

In this case, the pathology of the patient revealed tubular adenoma, which could be the result of chronic ulcer and inflammatory hyperplasia. Because of the advanced age of the patient, the endurance capacity for surgery was too limited, together with the canceration rate of tubular adenoma was low, PRP treatment was chosen with informed consent and prudence. There is no doubt that a satisfying result has been obtained in this case, but continuous follow-up of this patient needs to be performed and clinical trials with more samples are needed. Study on this issue is rare at present, and further experimental researches are needed to verify the hypothesis.

Up till now, the application of PRP in perianal fistulas has already been reported,^[20,21]^ but cases of PRP treatment for rectal ulcers have not been uncovered. A good result was acquired in this case, suggesting that the utilization of PRP may contribute to the creation of individualized treatment programs for patients with unhealed rectal ulcers in the future.

## Author contributions

Gengjun Liu wrote the main manuscript text and prepared figures 1–3, and Haiyan Wang put forward some important idea and suggestions. All authors reviewed the manuscript.

## Acknowledgments

This work was supported by the National Natural Science Foundation of China (Grant No. 21675074), the Natural Science Foundation of Shandong (ZR2017MH042), and the Clinical Medicine + X Research Project of Qingdao University (3716). We thank all the patients and colleagues who contributed to the study.
